# Leveraging geospatial technology for effective health planning for maternal and child health in Nigeria

**DOI:** 10.1093/oodh/oqaf021

**Published:** 2025-09-13

**Authors:** Amina Aminu Dorayi, Sakina Amin Bello, Hafiz Saidu Yaro, Hauwa Usman, Olufemi Ibitoye, Praise Agbe, Ayobami Afape, Ayuba Utsahyel Hildi, Liz Futrell, Masduk Abdulkarim, Marc Levy, Johanna Snell, Olena Borkovska, Nuradeen Maidoki, Fatima Tafoki, Folarin Akinsomi, Olubayo Adekanmbi, Chinazo Anebelundu

**Affiliations:** Department of Programs, Pathfinder International, Abuja, Federal Capital Territory 900231, Nigeria; Department of Programs, Pathfinder International, Abuja, Federal Capital Territory 900231, Nigeria; Department of Programs, Pathfinder International, Abuja, Federal Capital Territory 900231, Nigeria; Department of Programs, Pathfinder International, Abuja, Federal Capital Territory 900231, Nigeria; Department of Programs, Pathfinder International, Abuja, Federal Capital Territory 900231, Nigeria; Department of Programs, Pathfinder International, Abuja, Federal Capital Territory 900231, Nigeria; Department of Programs, Pathfinder International, Abuja, Federal Capital Territory 900231, Nigeria; Department of Programs, Pathfinder International, Abuja, Federal Capital Territory 900231, Nigeria; Pathfinder International, Washington, DC 20005, United States; Bill & Melinda Gates Foundation, Abuja, Federal Capital Territory 904101, Nigeria; GRID3, New York, NY 10017, United States; GRID3, New York, NY 10017, United States; GRID3, New York, NY 10017, United States; Natview Foundation for Technology Innovation, Abuja, Federal Capital Territory 900108, Nigeria; Natview Foundation for Technology Innovation, Abuja, Federal Capital Territory 900108, Nigeria; Natview Foundation for Technology Innovation, Abuja, Federal Capital Territory 900108, Nigeria; Data Science Nigeria, Lagos, Lagos State 100001, Nigeria; Data Science Nigeria, Lagos, Lagos State 100001, Nigeria

**Keywords:** geospatial, microplanning, digital health, health planning, maternal and child health

## Abstract

Nigeria faces persistent challenges in maternal and child health, with some of the highest mortality rates globally. Despite efforts to expand health care access, gaps remain due in part to inefficient data systems and reliance on paper-based microplanning. The GeoST4R project aimed to address these challenges by integrating geospatial technology into microplanning. In 2024, GeoST4R piloted the Geospatial Microplanning Toolkit (GMT)—a mobile application that enables health workers to visualize, update and analyze spatial data for targeted interventions—in Kano and Kaduna states. From harmonizing data sources (including health facilities, settlements and infrastructure) to building capacities of health workers to use the Toolkit, this article highlights the Toolkit development and implementation process, lessons learned and recommendations for scaling and sustaining digital microplanning solutions to improve health outcomes across Nigeria. GeoST4R trained 275 health workers and engaged stakeholders in hands-on sessions to update baseline data, map outreach sites and refine catchment areas. This led to significant improvements in health data, including the addition of 922 new settlements, updates to 616 settlement names and revisions of 73 facility names. For the first time, 1008 outreach sites were mapped, enhancing service coverage and data integration. The intervention also contributed to improve maternal and child health outcomes, boosting contraceptive uptake, skilled birth attendance and immunization coverage, demonstrating the value of geospatial tools for better health planning.

## INTRODUCTION

With the fourth highest maternal mortality ratio and the second highest neonatal mortality rate in the world, maternal and child health (MCH) remains a significant public health challenge in Nigeria [1]. Between 2000 and 2020, more than 25% of global maternal deaths occurred in Nigeria. Among the greatest challenges is that fewer than half of childbirths in Nigeria occur in health facilities [2]. Compounding this challenge is that many health planning processes remain paper-based rather than digital [3, 4].

In 2005, Nigeria adopted the WHO Reaching Every District strategy to improve immunization coverage [5]. The strategy addresses common obstacles by strengthening outreach services, enhancing supportive supervision, linking services with communities, monitoring and use of data for action and planning and management of resources. Nigeria has since tailored this strategy to create Reaching Every Ward (REW) to strengthen ward-level provision of quality services [6]. REW emphasizes mapping health services, optimizing logistics and ensuring efficient use of resources by using Geographic Information Systems (GISs) to map health services and streamline coordination among state and local governments and health workers to improve planning and monitoring. GIS can identify areas lacking access and plan focused interventions to reduce maternal and child mortality [7]. In Nigeria, where health disparities are pronounced, GIS tools offer a valuable means of bridging service gaps [8].

Microplanning, an essential component of REW, involves mapping health facility catchment areas, population estimates and service coverage gaps. The plans identify priority areas in need of intervention and facilitate the development of targeted work plans to deliver required services [9]. Despite the integration of GIS into health programs in Nigeria; however, many microplans are paper based, hindering the efficiency and timeliness of data collection, reporting and decision making. The slow pace of manual planning, the lack of real-time data and inaccuracies resulting from outdated data on population estimates, facility catchment areas and operational boundaries make it difficult to address urgent health needs [10].

### GEOST4R

Integrating digital tools into microplanning can enhance the accuracy of data, streamline decision-making and improve resource allocation. The Kano, Kaduna, Lagos and Gombe state governments, through their respective State Primary Health Care bodies, and in collaboration with the Geospatial Data/Tools/Technology for Reproductive, Maternal, Newborn and Child Health and Nutrition (RMNCH-N) Microplanning and Decision Support (GeoST4R) project, sought to enhance microplanning and decision making using geospatial data and tools. GeoST4R, funded by the Gates Foundation and led by Pathfinder International in partnership with Data Science Nigeria, Natview Foundation for Technology Innovation and GRID3, draws on successful approaches from polio immunization and the COVID-19 response to improve RMNCH-N services in Nigeria using innovative digital strategies and geospatial technology. GeoST4R strives to make quality data available to decision makers through a centralized database.

Using routine immunization as the entry point to primary health care, GeoST4R piloted the GMT in two of its four implementation states (Kano and Kaduna). This case study shares the implementation process, highlights key lessons and discusses the next steps for refining and scaling these tools.

## The GMT

To strengthen microplanning in Nigeria, GeoST4R collaborated with Novel-T, the Center for Integrated Earth System Information at Columbia University and Nigeria’s National Primary Health Care Development Agency (NPHCDA) to develop the GMT to address three health delivery and microplanning challenges: ineffective microplanning, lack of scalable data solutions and limited data sharing and integration.

The mobile GMT application allows health staff to record, edit and visualize spatial data and other essential information on an interactive user interface. The GMT database contains data on population, settlements, health facilities, points of interest and ward boundaries. Users can review and update and provide additional data. The GMT combines this information in a map and tables for export and use in microplanning.

### Creation of the GMT

**Figure 1:**
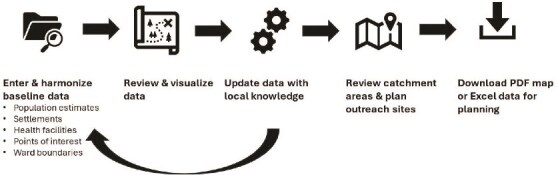
Process for using the geospatial microplanning toolkit

GeoST4R developed an Application Programming Interface (API) portal to create a hub for RMNCH-N, population and spatial data. GeoST4R then integrated, cleaned and standardized data sources; ensured security through authentication and authorization mechanisms and conducted usability and performance testing. Finally, GeoST4R created detailed API documentation to support user adoption and conducted security testing to ensure robustness against potential threats (Fig. 1).

#### Input data sources

Through coordination with government stakeholders, knowledge acquired from the environmental scan reports and availability of open operational geospatial data products, the following input data sources were used for the harmonization and data matching process (Table 1).

**Table 1:** Input data sources

**Data category**	**Data source**
Health facilities	Health and Education Facilities Analytics platform (2018) National Health Facility Registry (2023) eHealth Polio data distributed by GRID3 (2020) State Primary Health Care Development Management Board (2023) WHO Integrated Supportive Supervision (2023) Health facility baseline survey (2023) by Pathfinder
Settlements	Integrated REW Microplan Settlement (2023) Tabular List State Master List of Settlement (2023) Tabular List eHealth Polio data with location distributed by GRID3 (2020)
Points of interest	GRID3 Infrastructure Survey data conducted by Kaduna State Bureau of Statistics (motor parks and water points for Kaduna State) Kaduna Annual School Census conducted by the Kaduna Bureau of Statistics (2023) eHealth Polio school data with location distributed by GRID3 (2020)
Other core spatial data	Administrative boundaries and high-resolution population data (GRID3 data repository) Road data (obtained from OpenStreetMap and merged with Facebook Road data)

#### Data harmonization

Data harmonization yields a set of standardized core spatial data sets, organized by data type for each state and designed for interoperability with geospatial software and tools. The harmonized spatial layers ensure that all the data are consistent in format and attributes, each record has a corresponding source information, and a unique identifier code is assigned to each record. (This allows for future updates of the data set with additional information.) Data harmonization addresses gaps, filling in missing information where possible to create a more comprehensive dataset. The process involved three steps: (1) data pre-processing (cleaning and standardization); (2) checking spatial quality and assessing locations; and (3) spatial harmonization in cases where similar records appear in multiple sources with slight variations in location. Importantly, the harmonization process was not contingent on government input; instead, it relied on existing data sources and geospatial analysis to make informed decisions regarding the most accurate geographic coordinates.

The following geospatial data products were delivered for the implementation states:

- Harmonized health facilities, settlement names and locations
- Settlement extents with estimated total population and 5-year age groups
- Operational administrative boundaries at ward, Local Government Area (LGA) and state levels
- Points of interest, including schools, markets, mosques, churches (water points for Kaduna and Lagos and motor parks for Kaduna only)
- Road data
- High-resolution gridded population raster, total population and 5-year age groups

In addition to the geospatial data layers, health facility and settlement data sets were exported in a CSV format to allow for tabular validation of the lists using a spreadsheet or word-processing software. Upon harmonization, it was evident that validation from LGA teams and Ward Focal Persons (WFPs) were needed to produce a final geospatial dataset, as local knowledge was needed to make the final decisions on the health facility and settlement name records. The output files for health facilities and settlements (both spatial and tabular) include a column indicating if a health facility or settlement name record was matched, needs validation or remains unmatched.

### Pilot of the GMT

To assess the usefulness and feasibility of the GMT in supporting routine immunization, GeoST4R piloted the tool with health officials from 91 wards in Kano and Kaduna states (Table 2) between April and July 2024. The eight LGAs were selected in coordination with the State Emergency Routine Immunization Coordination Centre in Kano and Kaduna, so that areas with the highest number of zero-dose cases were included and both urban and rural wards were represented. Across the 91 wards, 545 people participated. The pilot had three objectives:

1. Evaluate the functionality and usability of the GMT and its outputs.
2. Assess the GMT support of key elements of microplanning for routine immunization; and
3. Identify requirements for continuing GMT use.

**Table 2:** Pilot participants

**State**	**LGA**	**Wards**	**Health facilities**	**Participants**
Kano	Dawakin Tofa	11	46	70
	Gabasawa	11	32	43
	Dambatta	10	40	60
	Dawakin Kudu	15	26	55
	Ungogo	11	44	63
	Gaya	10	32	58
Kaduna	Chikun	12	58	100
	Giwa	11	74	96
**Total**		**91**	**352**	**545**

#### Pilot activities

In early 2024, GeoSt4R engaged the Nigerian government at the national, state and local levels to ensure buy-in and then implemented the pilot in three phases: (1) training, (2) practical application and (3) exporting GMT outputs as PDF files or spreadsheets to the GeoST4R-provided tablets.

#### Phase 1: Training

Between April and June 2024, GeoST4R conducted four GMT trainings for 275 participants from state, LGA and ward-level teams in Kano and Kaduna. A core state project team, comprising key government stakeholders, was trained through a two-day training-of-trainers model covering GMT orientation, hands-on practice and troubleshooting techniques. Ward- and LGA-level teams received similar training over three days, with the final day dedicated to extended practical exercises.

To support the rollout, GeoST4R procured and configured 91 tablets (one per ward), pre-installed the GMT app and provided printed manuals to ensure hands-on learning. Additionally, GRID3 engaged trainers who completed intensive preparation through multiple interactive sessions over three weeks, deepening their technical knowledge and facilitation skills to cascade the training effectively.

#### Phase 2: Working sessions

After the training, GeoST4R trainers engaged two or three pilot wards at a time in 2-day working sessions at LGA headquarters. During the working sessions, users reviewed and updated baseline data in the GMT; mapped outreach locations and reviewed and adjusted catchment areas generated by the GMT.

On average, the 91 wards sent 6 people per ward to these working sessions, totaling 545 participants from 352 health facilities. These included the 275 trainees, plus facility officers-in-charge and ward heads who could provide additional information about settlements and their boundaries and health facilities and their operations. Each ward team included a WFP, a Ward Technical Officer, a ward head (often a traditional or community leader) and at least one representative from each health facility.

Ward teams brought their REW microplans, last updated in January 2024, to the working sessions, which aimed to harmonize data between the REW microplans and the GMT and identify updates needed in both.

#### Phase 3: GMT outputs

**Figure 2:**
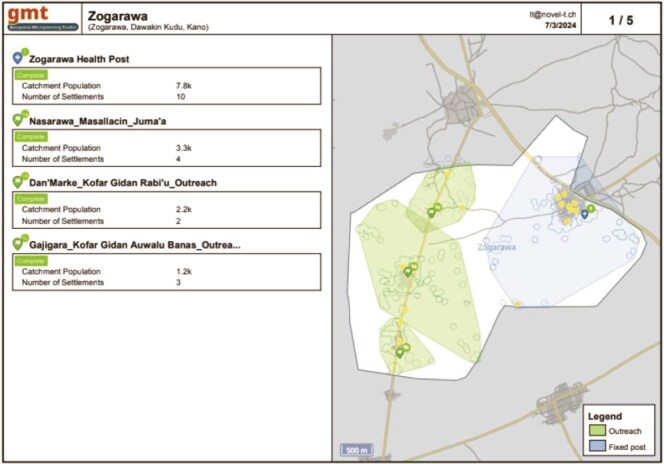
GMT PDF map export of Zogarawa Ward, Dawakin kudu LGA in Kano

After the working sessions, ward teams exported outputs from the GMT that were reviewed jointly by the GeoST4R team. The primary GMT outputs constitute PDF maps and data tables, but there is also an option to export a geodatabase (.gdb file) with all data for selected ward(s) (Fig. 2).

#### GMT pilot performance

To assess data improvements, GeoST4R exported the GMT database before the pilot began (baseline data) and compared it to the database at the end of the pilot. After the pilot, the participating wards kept the tablets with the GMT application for continued use. GeoST4R administered a post-pilot survey between August and September 2024 to all participating wards to collect information on whether they continued to use the GMT and had independent capacity to integrate GMT outputs in their workflows. The survey received 91 responses, one from each pilot ward in Kano and Kaduna.

### Data improvements

The GMT enabled improvements to baseline data through its easy-to-use features for visualizing, editing and adding or updating key data points (Table 3). The most significant changes included:

- 922 settlements added
- 327 old settlements deactivated
- 616 settlement names and 73 health facility names updated
- 1008 outreach sites mapped for the first time

**Table 3:** Select data improvements enabled by the GMT

**Updates enabled by GMT**	**Kano**	**Kaduna**
Settlement location updated	37	16
Settlement name updated	291	325
Settlement categorized as hard to reach	106	376
Settlement abandoned/no longer exists	157	167
Settlement linked to a different ward	37	11
Health facility location updated	5	13
Health facility name updated	20	53
Health facility linked to a different ward	0	2

### Post-pilot survey results

The survey revealed that most pilot wards have updated or plan to update their REW microplans using the GMT and have continued using the GMT in the months after the pilot. Of 91 respondents, 77 (85%) reported still using the GMT outputs and 76 (84%) were still using the GMT application. The GMT has been particularly useful for outreach session planning. Many pilot wards reported using it beyond routine immunization for other primary health care activities.

Of the 91 respondents, 39 (43%) had used the GMT to update hard copy microplans at the health facilities where they work. Thirty-four respondents (37%) had not updated their hard- or soft-copy REW microplans but planned to update their microplans between September and December 2024 (except for two who did not have a date scheduled). Of those who had not yet updated their microplans, all planned to use the GMT and its outputs to make changes to their microplans. Those who had updated their microplans using the GMT or intended to do so by December 2024 reported that the GMT enabled them to make several important changes to their microplans (Table 4).

**Table 4:** Select independent REW microplan updates made or planned after the pilot (*n* = 73)^a^

Baseline data improvements	Percent (%)
Added settlement(s) that were not previously on the primary health care REW microplan	64
Updated health facility catchment area(s) population	60
Updated settlement(s) population	53
Changed status (operating routine immunization or not) of health facility	33
Strategy improvements	
Changed location of outreach site(s)	55
Changed number of outreach site(s)	51
Changed strategy of settlement(s)	51
Added additional strategies to settlement(s)	45
Resources and scheduling improvements	
Changed service provision schedule	22
Changed required personnel needed for the facility	16
Changed required vaccinations or medications needed for the facility	15
Changed required equipment needed for the facility	3

^a^73 out of 91 respondents have made changes or plan to make changes to their microplan based on the GMT.

However, some wards had not integrated the GMT into their microplanning processes. Of the 91 WFPs, 17 (19%) reported that they had completed updates to their microplans but did not use the GMT or its outputs to inform them of their changes. When asked why they had updated their microplans without the GMT, respondents stated that it was either because they had not received the directive from their supervisors to do so, or because they did not fully understand how to incorporate the GMT into microplanning efforts.

Preparation for outreach sessions was among the most common uses of the GMT. Of WFPs, 61% reported that they used the GMT to review information before outreach sessions to identify the outreach catchment target populations; review specific settlements covered by the outreach site and their locations and determine the appropriate number of vaccinations and commodities needed for sessions. Additionally, 31 respondents (34%) reported holding an outreach session at a different location based on the information from the GMT. WFPs have held outreach sessions at 77 new outreach sites.

Respondents also reported using the GMT as a guide; a monitoring tool for planning and reviewing routine immunization sessions; and a record of health facility and ward information. In addition to routine immunization, 19 WFPs (21%) mentioned using the GMT to plan other primary health care services: polio, measles, meningococcal, malaria, Antenatal Care (ANC), growth monitoring, maternal and child nutrition, family planning, non-invasive prenatal diagnosis, dental and pharmaceutical services and curative care.

### Barriers to continued use of the GMT

Although independent use of the GMT was high, 15 WFPs (16%) reported discontinuing use. Common reasons included a lack of planned activities (e.g. microplanning had not begun), insufficient guidance after the pilot or the perception that the tool was not relevant to their current tasks.

In the post-pilot survey, 50 out of 91 (55%) WFPs reported having no issues using the GMT; however, the remaining 45% reported encountering difficulties with at least one function. Splitting/merging settlements and editing ward boundaries were considered the most difficult features. Nearly half (48%) of survey respondents experienced other difficulties that restricted their ability to use the GMT, including challenges understanding exported data, integrating the GMT outputs with existing microplans and accessing a tablet.

To enhance the adoption of the GMT, 58 WFPs (64%) expressed a need for additional support, including training on specific features like locating outreach sites, creating custom catchment areas and exporting data. Respondents requested additional technical support when new features are added to the GMT, as well as regular “refresher” training on the tool.

Finally, 34 WFPs (37%) of respondents reported challenges related to network connectivity, technical issues and comfort with independent use. Overcoming these barriers is crucial for ensuring the long-term sustainability of the GMT.

### Improvements in maternal and child health data

GeoST4R collected MCH data from the District Health Information Software 2 at baseline, midline and endline during the GMT pilot in Kano and Kaduna States. The findings showed notable improvements across key indicators, largely attributed to enhanced planning and data use enabled by the GMT.

#### Modern Contraceptive Uptake

**Figure 3:**
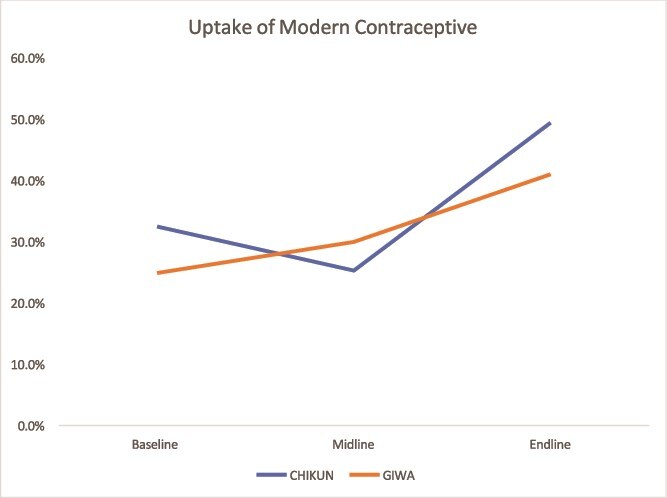
Trend in the uptake of modern contraceptives in Kaduna

**Figure 4:**
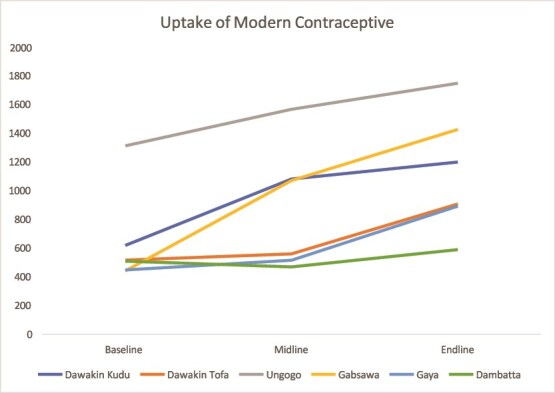
Trend in the uptake of modern contraceptives in Kano

Both states recorded increases in contraceptive use. In Kaduna, uptake in Giwa rose from 24.9% to 41% and in Chikun from 32.5% to 49.4%. Kano also showed steady gains across most LGAs (Figs 3 and 4).

#### ANC attendance

**Figure 5:**
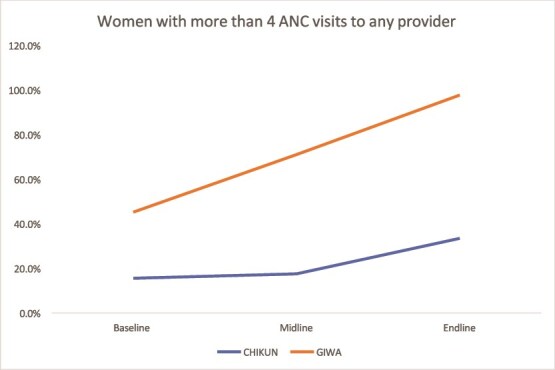
Trend in the 4+ ANC visit in Kaduna

**Figure 6:**
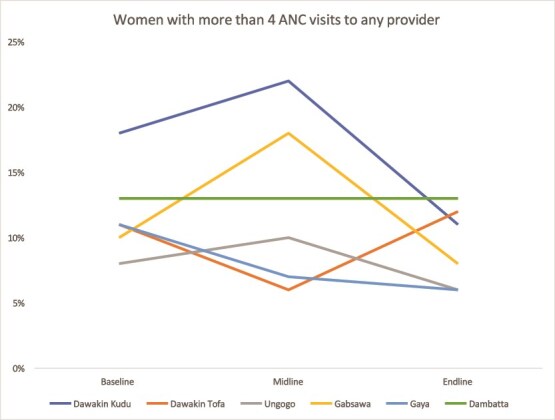
Trend in the 4+ ANC visit in Kano

ANC attendance (four or more visits) improved significantly in Kaduna, with coverage rising from 15.6% to 33.5% in Chikun and from 45.3% to 97.8% in Giwa. Kano’s performance was mixed, with some LGAs improving while others stagnated or declined (Figs 5 and 6).

#### Skilled birth attendance

**Figure 7:**
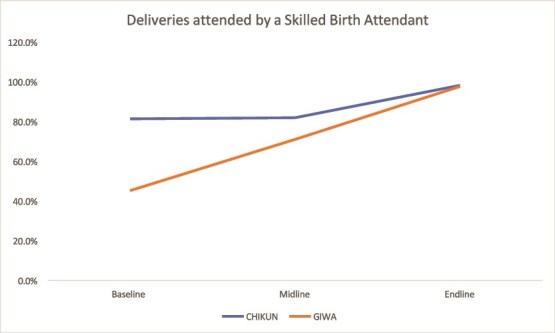
Trend in skilled birth attendance in Kaduna

**Figure 8:**
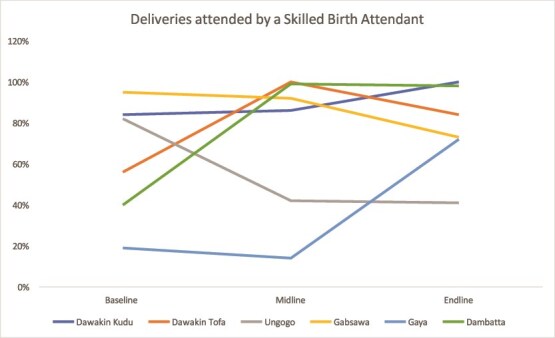
Trend in skilled birth attendance in Kano

Both states achieved strong progress. Kaduna’s pilot LGAs surpassed 98% skilled birth attendance by endline, while Kano’s LGAs, including Dawakin Kudu and Dambatta, approached full coverage (Figs 7 and 8).

#### Penta 3 immunization coverage

**Figure 9:**
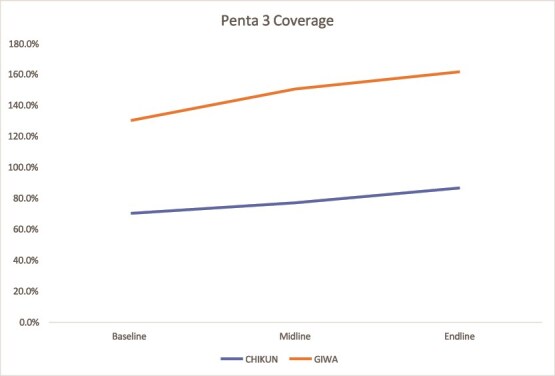
Trend in Penta 3 coverage in Kaduna

**Figure 10:**
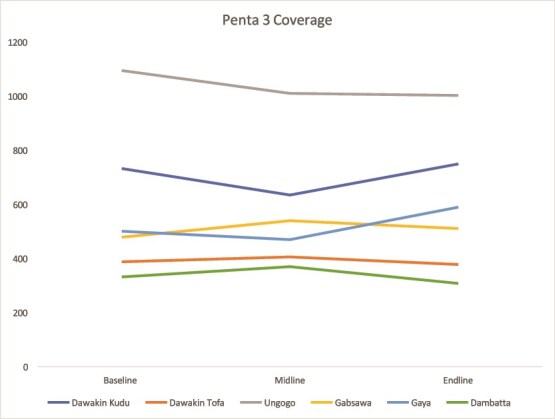
Trend in Penta 3 coverage in Kano

Immunization rates improved steadily in Kaduna across both LGAs. In Kano, progress was uneven, with modest gains in some areas and stagnation in others, highlighting the need for targeted immunization efforts (Figs 9 and 10).

These results demonstrate that geospatial planning through GMT can contribute to measurable improvements in MCH service coverage, while also revealing areas requiring intensified support.

### Lessons learned

1. The GMT pilot demonstrated that geospatial technology is a practical, scalable solution for strengthening microplanning in Nigeria, replacing outdated paper-based systems with accurate digital maps and high-resolution satellite imagery to support better planning and decision-making. The positive results from this pilot make a strong case for national adoption to improve immunization microplanning.
2. To enhance usability, GeoST4R procured 91 affordable tablets with larger screens, as most frontline users in pilot wards only owned basic feature phones unsuitable for the tool which only operates on smartphones. For future scale-up, states should allocate dedicated funding for internet subscriptions within their annual budgets and integrate these costs into Basic Health Care Provision Fund business cases to ensure uninterrupted access.
3. Effective and sustained use of the GMT requires ongoing technical support beyond initial training. We recommend that states develop a clear, user-friendly standard operating procedure that integrates GMT into routine immunization workflows, defines roles and aligns with government systems. To reinforce learning, visual job aids such as shareable videos, posters or flowcharts should be displayed in health facilities as daily reminders on when and how to apply the tool. In addition, regular mentoring and quarterly pause-and-reflect sessions in priority wards will help address persistent challenges, particularly in interpreting data or updating microplans. These sessions can create space for peer learning, hands-on problem-solving and timely adaptation.
4. Sustained government leadership, resourcing for internet connectivity and maintenance of devices will be essential for institutionalizing GMT. The improvements in microplanning data quality observed during this pilot provide a compelling case for investing in geospatial tools and other innovations to improve microplanning for MCH services in Nigeria.

## Data Availability

Data available upon request.

## References

[1] World Health Organization Regional Office for Africa (WHO AFRO). Maternal mortality: The urgency of a systemic and multisectoral approach in mitigating maternal deaths in Africa. In Integrated African Health Observatory. Brazzaville: World Health Organization, Regional Office for Africa, 2023. https://files.aho.afro.who.int/afahobckpcontainer/production/files/iAHO_Maternal_Mortality_Regional_Factsheet.pdf

[2] Cresswell J . Trends in Maternal Mortality 2000 to 2020: Estimates by WHO, UNICEF, UNFPA, World Bank Group and UNDESA/Population Division. 1st edn. Geneva: World Health Organization, 2023.

[3] Uzochukwu BS, Etiaba E, Ezumah N et al. Quality of maternal and child health data within the Health Management Information System in Nigeria: A post field reflection [Policy brief]. REVAMP project, University of Nigeria Enugu Campus, 2017. 10.13140/RG.2.2.22955.75047

[4] Tijani B, Jaiyeola T, Oladejo B et al. Improving data integrity in public health: a case study of an outbreak management system in Nigeria. *Global Health: Science and Practice* 2021;9:S226–33. 10.9745/GHSP-D-21-00240 34845046 PMC8628498

[5] Reaching Every District (RED), 2017 Revision. Brazzaville, Republic of the Congo: World Health Organization, 2017.

[6] John Snow, Inc. (JSI). Making “Reaching Every Ward” operational: A step toward revitalizing primary health care in Nigeria. Abuja, Nigeria, 2009.

[7] Alliance for Malaria Prevention. Case Study: Geospatial Tools for Microplanning in Nigeria. Abuja, Nigeria: Alliance for Malaria Prevention, 2023. Retrieved from https://allianceformalariaprevention.com/resource-library/resource/case-study-geospatial-tools-for-microplanning-nigeria/

[8] Biu PW et al. GIS in healthcare facility planning and management: a review. *World J Adv Res Rev* 2024;21:012–9. 10.30574/wjarr.2024.21.1.2682

[9] Microplanning: Evidence on Pro-Equity Interventions to Improve Immunization Coverage for Zero-Dose Children and Missed Communities. Vol. 360. Durham, NC, USA: FHI, 2023.

[10] Umeh GC, Madubu DM, Korir C et al. Micro-planning for immunization in Kaduna state, Nigeria: lessons learnt, 2017. *Vaccine* 2018;36:7361–8. 10.1016/j.vaccine.2018.10.020 30366806 PMC6238078

